# Impacts of Various Straw Mulching Strategies on Soil Water, Nutrients, Thermal Regimes, and Yield in Wheat–Soybean Rotation Systems

**DOI:** 10.3390/plants14142233

**Published:** 2025-07-19

**Authors:** Chaoyu Liao, Min Tang, Chao Zhang, Meihua Deng, Yan Li, Shaoyuan Feng

**Affiliations:** College of Hydraulic Science and Engineering, Yangzhou University, Yangzhou 225009, China

**Keywords:** straw mulching, wheat–soybean rotation, mulching timing, mulching thickness, planting density, soil moisture–fertility–temperature regime, crop yield, structural equation modeling

## Abstract

Straw mulching is an important strategy for regulating soil moisture, nutrient availability, and thermal conditions in agricultural systems. However, the mechanisms by which the mulching period, thickness, and planting density interact to influence yield formation in wheat–soybean rotation systems remain insufficiently understood. In this study, we systematically examined the combined effects of straw mulching at the seedling and jointing stages of winter wheat, as well as varying mulching thicknesses and soybean planting densities, on soil properties and crop yields through field experiments. The experimental design included straw mulching treatments during the seedling stage (T_1_) and the jointing stage (T_2_) of winter wheat, with soybean planting densities classified as low (D_1_, 1.8 × 10^5^ plants·ha^−1^) and high (D_2_, 3.6 × 10^5^ plants·ha^−1^). Mulching thicknesses were set at low (S_1_, 2830.19 kg·ha^−1^), medium (S_2_, 8490.57 kg·ha^−1^), and high (S_3_, 14,150.95 kg·ha^−1^), in addition to a no-mulch control (CK) for each treatment. The results demonstrated that (1) straw mulching significantly increased soil water content in the order S_3_ > S_2_ > S_1_ > CK and exerted a temperature-buffering effect. This resulted in increases in soil organic carbon, available phosphorus, and available potassium by 1.88−71.95%, 1.36−165.8%, and 1.92−36.34%, respectively, while decreasing available nitrogen content by 1.42−17.98%. (2) The T_1_ treatments increased wheat yields by 1.22% compared to the control, while the T_2_ treatments resulted in a 23.83% yield increase. Soybean yields increased by 23.99% under D_1_ and by 36.22% under D_2_ treatments. (3) Structural equation modeling indicated that straw mulching influenced yields by modifying interactions among soil organic carbon, available nitrogen, available phosphorus, available potassium, bulk density, soil temperature, and soil water content. Wheat yields were primarily regulated by the synergistic effects of soil temperature, water content, and available potassium, whereas soybean yields were determined by the dynamic balance between organic carbon and available potassium. This study provides empirical evidence to inform the optimization of straw return practices in wheat–soybean rotation systems.

## 1. Introduction

Global population growth and escalating pressure on arable land are subjecting agricultural production to unprecedented challenges. In this context, enhancing agricultural productivity and fostering sustainable development have emerged as central priorities in global agricultural research [1]. Effective soil health management is widely recognized as essential to achieving these objectives, particularly in addressing issues such as climate change, soil degradation, and ecological damage. Consequently, improving soil quality has become a critical focus of contemporary research [2,3].

Straw, a biomass byproduct generated during crop production, has been widely recognized as an effective resource for reuse [4]. Within the domain of soil health management, straw mulching is regarded as an environmentally sustainable agricultural practice, which is particularly valuable in mitigating the negative impacts of soil degradation, drought, and climate change. Globally, the application of straw mulching has demonstrated significant agronomic benefits across diverse cropping systems, including improvements in soil physical properties, the enhancement of water retention capacity, the enrichment of soil fertility, the regulation of soil temperature, reductions in erosion, and the mitigation of extreme weather impacts on crop performance [5,6]. Consequently, an increasing number of countries are incorporating straw mulching into their strategic frameworks for sustainable agricultural development. Numerous studies have demonstrated that straw mulching has multifaceted effects on the soil–plant system. With respect to soil properties, straw mulching enhances soil structure, reduces bulk density, increases soil organic matter content, improves water-holding capacity, and stabilizes soil temperature regimes [7,8]. These improvements foster a more favorable environment for root development and microbial activity. In terms of plant growth, straw mulching reduces evaporative stress, promotes root development, and facilitates more efficient nutrient uptake, collectively supporting improved physiological performance [9,10]. As a result, these enhancements at both the soil and plant levels contribute to increased crop productivity, including higher grain yields, improved yield components, and greater resource use efficiency [11,12].

Straw mulching also plays a critical role in the cultivation of major grain crops such as wheat and soybeans. Winter wheat, a staple food crop cultivated globally, is highly sensitive to variations in soil water, temperature, and nutrient availability throughout its growth cycle—factors that are particularly influential in regions characterized by drought and large temperature fluctuations. Appropriately applied straw mulching has been shown to enhance the crop’s resilience and improve yields [13]. Similarly, soybeans, a key oilseed crop, contribute significantly to the global food supply and support soil health through their nitrogen-fixing capabilities [14]. Furthermore, the wheat–soybean rotation system, a common agricultural practice, has been shown to improve soil structure and fertility, thereby enhancing long-term land productivity [15]. In this system, the alternating planting cycles of wheat and soybeans leverage their complementary biological characteristics: wheat requires higher soil moisture, while soybeans enrich the soil with nitrogen [16,17]. Therefore, the implementation of appropriate straw mulching in a wheat–soybean rotation system can enhance crop resilience to environmental stressors and boost yields. Accordingly, it can further promote soil quality, reduce the risks of erosion and degradation, and offer valuable insights for advancing sustainable agricultural development worldwide.

Regional agricultural practices have demonstrated that the effectiveness of straw mulching is influenced not only by the thickness of the mulch layer but also by factors such as the timing of applications, the crop growth stage, and the planting density [18,19]. In particular, against the backdrop of intensified global climate change and an increasing frequency of extreme weather events, there is a pressing need to scientifically optimize straw mulching practices—including the mulch thickness and initiation timing—to accommodate varying planting densities and environmental stresses across regions with diverse climatic conditions and soil types. The underlying mechanisms involved, such as water–heat–nutrient dynamics and crop responses, remain insufficiently understood. Addressing these gaps is a key challenge for maximizing the potential of straw mulching technologies. The core research questions of this study are as follows: In a winter wheat–soybean rotation system, how do different straw mulch thicknesses and initiation timings affect the soil moisture, temperature, and nutrient status? And how do these changes, in turn, influence the growth physiology and final yield of winter wheat and soybeans under varying planting densities?

Based on the aforementioned context and research questions, this study proposes the following hypotheses: (1) Compared with no mulching, an optimal straw mulch thickness will significantly improve the soil hydrothermal conditions (by increasing soil moisture and buffering temperature fluctuations) and enhance nutrient availability (by increasing organic matter and available nutrients), thereby promoting the growth and yield of winter wheat and soybeans. However, excessive mulching may have inhibitory effects, such as reducing soil temperature or increasing disease incidence. (2) Applying straw mulch prior to key crop growth stages (such as the pre-wintering/greening stage for winter wheat or the seedling stage for soybeans) will be more effective in optimizing the soil environment and alleviating stress than delayed mulching, resulting in greater yield benefits. (3) The yield-enhancing effects of straw mulching will be more pronounced at higher planting densities, indicating that mulching strategies (primarily in terms of thickness and initiation timing) should be optimized in conjunction with target planting densities to achieve optimal results.

This study aims to examine the effects of straw mulching on the growth and yield of winter wheat and soybeans within the framework of sustainable agricultural development. Specifically, it investigates how varying mulch thicknesses influence soil water, temperature, nutrient dynamics, and crop performance. Additionally, the study explores the optimization of straw mulching strategies based on different initiation times and planting densities, with the goal of developing broadly applicable and practical technical solutions for diverse agricultural regions. Notably, this study moves beyond the traditional approach of analyzing isolated soil factors or single crop response indices. Structural equation modeling (SEM) is employed to systematically analyze and quantify the interaction pathways among multiple key soil physicochemical parameters—including moisture, temperature, organic matter, and available nutrients—under different straw mulching thicknesses and initiation periods. Furthermore, the joint influence of these parameters on the yield and yield components of winter wheat and soybeans (e.g., number of spikes, thousand-grain weight, number of plants, hundred-seed weight) is explored. This methodology enables a comprehensive elucidation of the causal networks and relative contributions through which mulching practices enhance soil environments and, consequently, crop production. By providing a more mechanistic, quantitative, and predictive scientific basis for optimizing mulching models, this research offers an innovative aspect that distinguishes it from previous related work. Ultimately, the findings of this study are expected to provide novel perspectives and solutions for sustainable agriculture, promote the efficient utilization of agricultural resources, and contribute to ecological environmental protection.

## 2. Materials and Methods

### 2.1. Overview of the Study Area

**Figure 1:**
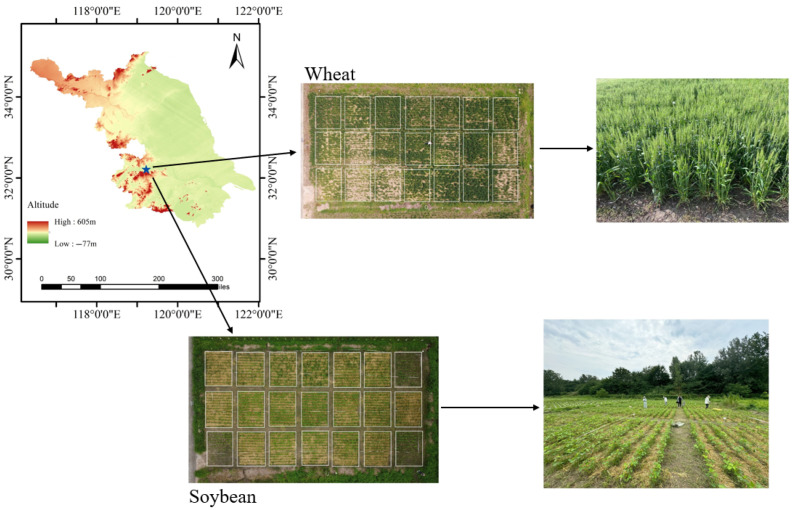
Geographic location of the study site for the wheat–bean rotation conducted during 2023–2024.

**Figure 2:**
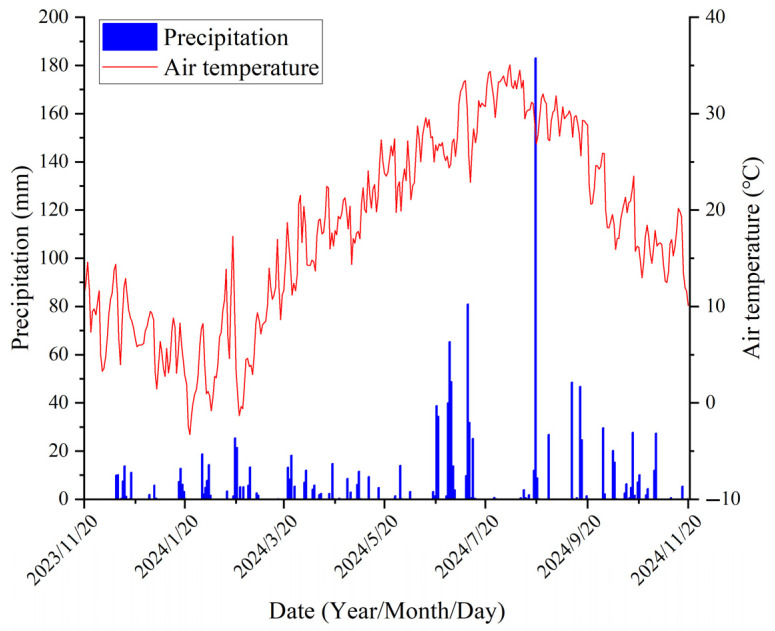
Variations in mean daily temperature and daily rainfall in the study area during the experimental period.

The experiment was conducted at the Agricultural Water, Hydrology, and Hydroecology Experimental Site of Yangzhou University, located in Yangzhou City, Jiangsu Province, China (32°21′ N, 119°24′ E, 5 m above sea level), from November 2023 to October 2024 (Figure 1). The study site is situated in the central Jianghuai Plain, a region characterized by a subtropical monsoon climate, with an average annual temperature of 14.8 °C and an average annual precipitation of 1063 mm. The soil at the experimental site is classified as loamy, with a bulk density of 1.43 g·cm^−3^. The initial soil properties for the 0−30 cm soil layer were as follows: organic carbon content of 5.21 g·kg^−1^, available nitrogen at 43.38 mg·kg^−1^, available phosphorus at 8.90 mg·kg^−1^, and available potassium at 57.33 mg·kg^−1^. The soil mechanical compositions consisted of 46.53% sand particles (0.02−2 mm), 44.72% silt particles (0.002−0.02 mm), and 8.75% clay particles (<0.002 mm). The precipitation distribution and air temperature fluctuations throughout the experimental period are illustrated in Figure 2.

### 2.2. Experimental Design

The experiment was conducted under a winter wheat–summer soybean rotation system, utilizing the main cultivars ‘Yangmai 33’ and ‘Ruidou 1’, commonly grown in Jiangsu Province. Winter wheat (*Triticum aestivum* L.) was sown in late November 2023 and harvested in late May 2024, while summer soybeans (*Glycine max* (L.) Merr.) were sown in late June 2024 and harvested in late October 2024. The winter wheat trial comprised seven treatments: straw mulch was uniformly applied at the seedling stage (T_1_) and the jointing stage (T_2_) with crushed wheat straw (5–8 cm in length) at thicknesses of 1 cm (S_1_: 2830.19 kg·ha^−1^), 3 cm (S_2_: 8490.57 kg·ha^−1^), and 5 cm (S_3_: 14,150.95 kg·ha^−1^), as well as a no-straw-returned control (CK). The summer soybean experiment employed two sowing densities (D_1_: 1.8 × 10^5^ plants·ha^−1^ and D_2_: 3.6 × 10^5^ plants·ha^−1^). Within each density, straw mulch treatments were established with thicknesses of 1 cm, 3 cm, and 5 cm, alongside a no-straw-returned control treatment. In this study, the straw utilized was locally sourced winter wheat straw. The primary organic constituents of the straw were cellulose, hemicellulose, and lignin, comprising approximately 33.41%, 30.40%, and 22.21% of the total composition, respectively. The carbon-to-nitrogen (C/N) ratio was 94:1. The straw contained approximately 0.4% nitrogen, 0.12% phosphorus, and 1.0% potassium.

Each treatment was replicated three times, resulting in a total of 21 plots arranged in a randomized block design. Each plot measured 20 m^2^ (5 m × 4 m), with a 1 m buffer zone between adjacent plots. A locally used compound fertilizer (N-P_2_O_5_-K_2_O: 15%-15%-15%) was applied uniformly at a rate of 420.00 kg·ha^−1^ across all plots. Throughout the experimental period, no irrigation was applied; the crops were grown entirely under natural rainfall conditions. Other field management practices, including the excavation of drainage ditches, weed control, and pest management, were conducted in accordance with local agricultural practices.

### 2.3. Measurement Indicators

#### 2.3.1. Soil Mass Water Content

During the growing periods of wheat and soybeans, soil samples were collected between crop rows at the center of each plot at depths of 10, 30, 50, 70, and 90 cm using a soil auger to determine the soil water content within the 0–100 cm profile. This depth range was selected because it encompassed the primary root growth zones of both wheat and soybeans [20,21]. In the wheat–soybean rotation system, crop roots exhibited strong water and nutrient uptake capacity within this depth range, making soil water content a critical factor influencing crop growth. By systematically monitoring the soil profile from 0 to 100 cm, the distribution characteristics of soil water were comprehensively reflected, thereby providing scientific evidence for evaluating the impact of management practices such as straw mulching on soil water regulation.

Sampling was conducted every 10 days throughout the experimental period. The wet weight of the soil samples from each plot was recorded immediately after collection. Subsequently, the samples were oven-dried at 105 °C for 8 h to determine their dry weight. The soil water content (***θ***, g·g^−1^) was determined using the gravimetric method and calculated according to the following formula:

(1) $$\theta =\frac{m_{wet}-m_{dry}}{m_{dry}}\times 100\%$$

where *m_wet_* is the mass of the field moist soil sample (g) and *m_dry_* is the mass of the oven-dried soil sample (g).

#### 2.3.2. Soil Temperature Measurement

Geothermometers were installed at depths of 5, 10, 15, 20, and 25 cm between crop rows at the center of each plot. Soil temperatures were recorded at 8:00 a.m., 2:00 p.m., and 8:00 p.m., with observations conducted once every seven days. Additionally, during the early, middle, and late periods of each month, soil temperatures across the profile were continuously monitored from 08:00 to 20:00 at two-hour intervals on representative sunny and cloudy days. Soil temperature was measured at a depth of 0–25 cm, as temperatures within this range significantly influenced the germination, emergence, and growth of wheat and soybeans. The root systems of both crops were most active within the 0–25 cm depth, and variations in the soil temperature directly affected root growth, nutrient uptake, and physiological activities [22,23]. Furthermore, soil temperature fluctuations below 25 cm were relatively moderate and had a smaller impact on crop growth [24]. By monitoring the soil temperature at the 0–25 cm depth, the real-time effects of temperature changes on crop growth were more accurately captured, providing a basis for optimizing management practices.

#### 2.3.3. Crop Dry Matter and Yield

Dry matter: Sampling for winter wheat was conducted at the regreening stage, jointing stage, flowering stage, grain-filling stage, and maturity stage. For soybeans, at the fourth trifoliate leaf stage, full-bloom stage, full-pod stage, full-seed stage, and full-maturity stage. From each plot, ten winter wheat plants and three soybean plants exhibiting uniform and representative growth were selected. The sampled plants were first oven-treated at 105 °C for 30 min to halt physiological activity, followed by drying at 80 °C until a constant weight was achieved. The dry weight was then recorded.

Yield: At crop maturity, yield components were measured by selecting a 1 m^2^ area within each plot. For winter wheat, the spike density, the grain number per spike, and the thousand-grain weight were determined. For soybeans, the number of plants per square meter, the number of pods per plant, and the hundred-grain weight were assessed. The final harvests of winter wheat and soybeans were performed separately for each plot to determine the total yield.

#### 2.3.4. Soil Nutrients

Soil samples from the 0−30 cm layer were collected from each treatment plot prior to sowing and at the harvest of both wheat and soybean. The 0–30 cm soil depth was selected for nutrient measurements because nutrients within this range most accurately reflected the nutrient supply required for the growth of wheat and soybeans. In the wheat–soybean rotation system, the majority of crop roots were distributed within the 0–30 cm soil layer, enabling a more precise assessment of nutrient uptake and utilization by crops [25]. Measuring soil nutrients at this depth allowed for a more accurate evaluation of the effects of different treatment practices on soil fertility, thereby providing a more scientific basis for crop management decisions.

The soil samples were air-dried naturally to remove impurities and then ground and passed through a 2 mm sieve for subsequent analysis. Soil organic carbon was determined using the potassium dichromate oxidation method. Soil available nitrogen was measured by the alkaline diffusion method with boric acid absorption. Soil available phosphorus was assessed by sodium bicarbonate extraction followed by the molybdenum antimony colorimetric method. Soil available potassium was measured using ammonium acetate extraction and the flame photometric method.

#### 2.3.5. Soil Bulk Density

The soil bulk density was measured using the cutting ring method at the end of the crop maturity period. Undisturbed soil samples were collected using a cutting ring with a diameter of 5 cm and a height of 5 cm. The samples were oven-dried at 105 °C until a constant weight was achieved, and the dry weight was recorded. The soil bulk density was then calculated using the following formula:

(2) $$\rho =\frac{M}{V}$$

where *ρ* is the soil bulk density (g·cm^−3^), *M* is the mass of the oven-dried soil sample (g), and *V* is the volume of the sampling ring (cm^3^).

#### 2.3.6. Coefficient of Variation

The coefficient of variation (*C_v_*) represents the relative dispersion of soil temperature data across different treatments or growth stages of wheat and soybeans. The calculation formula for the *C_v_* is as follows:

(3) $$C_{v}=\frac{\sigma }{\bar{X}}\times 100\%$$

where *σ* is the standard deviation of soil temperature under different treatments or growth stages, and $\bar{X}$ is the corresponding mean soil temperature.

#### 2.3.7. Structural Equation Modeling

Structural equation modeling (SEM) is a multivariate statistical analysis technique used to test complex networks of causal relationships among variables [26]. Compared with traditional regression analysis, SEM not only accommodates multiple dependent and independent variables but also enables the construction of latent variables to comprehensively reflect information from multiple indicators [27]. Moreover, it separates measurement errors, quantifies mediating effects, and assesses the theoretical validity of the overall model structure [28]. SEM is not only applicable to the social sciences but has also demonstrated robust analytical capabilities, gaining increasing importance in agriculture, particularly in areas such as agricultural ecology and resource management. Bocean employed SEM as the analytical tool to comprehensively assess the impact of digital technology on the sustainable food system [29]. Similarly, Liu et al. adopted an SEM framework to investigate rice yield formation under reduced nitrogen and a biochar amendment [30].

Drawing on previous related studies, SEM was used to analyze the comprehensive effects of various soil physicochemical parameters on wheat and soybean yields, as well as yield components, under different straw mulching treatments. In the present analysis, the measured soil parameters (e.g., soil water content, temperature, nutrients), yield compositions (e.g., spike density, grains number per spike, thousand-grain weight), and final yield were used as measurement indicators. The SEM framework was structured with soil- and plant-related variables as the first layer, yield components as the second layer, and yield as the third layer. Interactions among variables were represented using path diagrams. The primary indicators used to evaluate the model fit included (1) a chi-square-to-degrees-of-freedom ratio (χ^2^/df): a value less than 3.00 indicates a good model fit by balancing model complexity and avoiding overfitting. (2) A Root Mean Square Error of Approximation (RMSEA): a value less than 0.08 suggests a reasonable fit between the model and the data. (3) A comparative fit index (CFI): a value greater than 0.90 indicates a high degree of model fitness [31].

### 2.4. Data Processing

Data preprocessing was performed using Microsoft Excel 2024 (Microsoft Corporation, Redmond, WA, USA). Graphical representations were generated with Origin 2022 (OriginLab Corporation, Northampton, MA, USA), and statistical analyses were conducted using IBM SPSS Statistics 27.0 (IBM Corporation, Armonk, NY, USA). For soil physicochemical properties and crop yields across different treatments, means and standard deviations were calculated. Differences among treatments were assessed using Duncan’s new multiple range test, with the significance level set at *p* < 0.05. Structural equation modeling was conducted using AMOS 28.0 (IBM Corporation, Armonk, NY, USA) to elucidate the effects of soil physicochemical parameters on crop yields and their components.

## 3. Results

### 3.1. Effect of Straw Mulching on Soil Water Content

#### 3.1.1. Changes in Soil Profile Water Content Under Winter Wheat Cultivation

**Figure 3:**
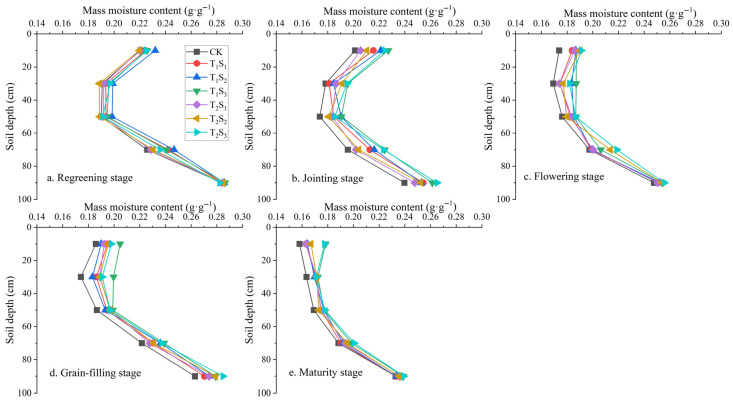
Distribution of soil water content in the 0−100 cm profile under different straw mulching treatments during the main growth stage of winter wheat. CK: no mulching; T_1_S_1_/T_1_S_2_/T_1_S_3_: 1/3/5 cm crushed straw mulch applied since seedling stage; T_2_S_1_/T_2_S_2_/T_2_S_3_: 1/3/5 cm crushed straw mulch applied since jointing stage.

The dynamics of soil water content at different soil depths exhibited a consistent pattern across all treatments, generally decreasing as wheat advanced through its reproductive stages (Figure 3). From the regreening to the grain-filling stage, the soil water content in the 0−100 cm profile of all treatments showed an initial decrease from the surface to 30 cm depth, followed by an increase with further depth. Specifically, in the 0–30 cm layer, the soil water content declined by 8.80% (CK), 10.22% (T_1_S_1_), 9.61% (T_1_S_2_), 7.88% (T_1_S_3_), 7.70% (T_2_S_1_), 8.56% (T_2_S_2_), and 8.79% (T_2_S_3_) from regreening to grain-filling. Below 30 cm, the water content progressively increased, with increments from 30 cm to 90 cm reaching 45.15% (CK), 44.21% (T_1_S_1_), 42.02% (T_1_S_2_), 38.91% (T_1_S_3_), 42.40% (T_2_S_1_), 43.57% (T_2_S_2_), and 42.27% (T_2_S_3_). At maturity, the soil water content continued to increase with depth across all treatments. The water content rise from 0 to 100 cm was 47.37% (CK), 42.91% (T_1_S_1_), 42.03% (T_1_S_2_), 33.85% (T_1_S_3_), 44.52% (T_2_S_1_), 40.97% (T_2_S_2_), and 34.74% (T_2_S_3_), indicating that deeper layers retained more moisture regardless of treatment.

Straw mulch treatments (T_1_S_1_–T_2_S_3_) consistently maintained higher soil water content than the control (CK), with the effect most pronounced in the 0−30 cm layer (Figure 3). During the wheat growth period, the average water content in this layer increased by 3.96% (T_1_S_1_), 5.43% (T_1_S_2_), 8.70% (T_1_S_3_), 3.54% (T_2_S_1_), 4.60% (T_2_S_2_), and 7.48% (T_2_S_3_) relative to the CK (*p* < 0.05). The T_1_S_3_ and T_2_S_3_ treatments, in particular, resulted in significantly higher water content than the CK from the jointing to grain-filling stages (*p* < 0.05; Figure 3). In contrast, the influence of straw mulching on the soil water content was less significant in the 30–100 cm soil layer. Increases compared to the CK were 3.34% (T_1_S_1_), 4.43% (T_1_S_2_), 5.77% (T_1_S_3_), 2.55% (T_2_S_1_), 3.35% (T_2_S_2_), and 5.90% (T_2_S_3_), with no statistically significant differences among treatments.

#### 3.1.2. Changes in Soil Profile Water Content Under Soybean Cultivation

**Figure 4:**
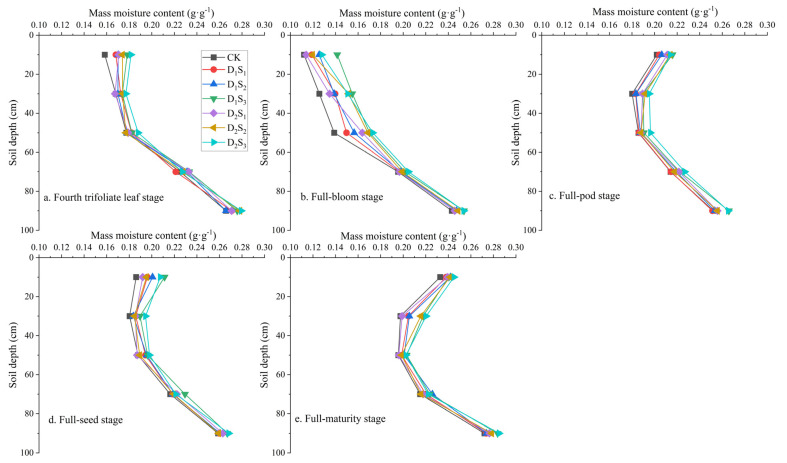
Distribution of soil water content in the 0−100 cm profile of soybeans under different straw mulching treatments across each growth stage. CK: no straw mulching treatment; D_1_S_1_/D_1_S_2_/D_1_S_3_: seeding density of 1.8 × 10^5^ plants·ha^−1^ with straw mulch thickness of 1/3/5 cm; D_2_S_1_/D_2_S_2_/D_2_S_3_: seeding density of 3.6 × 10^5^ plants·ha^−1^ with straw mulch thickness of 1/3/5 cm.

The soil water content across different depths exhibited consistent trends among treatments during the soybean growth period, characterized by a sequence of decrease→increase→decrease→increase across the stages from the fourth trifoliate leaf to full maturity (Figure 4). At the fourth trifoliate leaf, full-pod, full-seed, and full-maturity stages, the soil water content declined from the surface to 30 cm, followed by a marked increase from 30 cm to 100 cm. The reduction in water content between 10 and 30 cm ranged from 5.93% (CK) to 10.11% (D_1_S_3_), with the CK showing the smallest decrease and D_1_S_3_ the largest. Conversely, the soil water content from 30 to 90 cm increased by 42.96–45.17%, peaking in the CK treatment and being lowest in D_1_S_1_. During the full-bloom stage, the soil water content continuously increased with depth in all treatments. The 0–100 cm profile saw increases of 116.93% (CK), 106.43% (D_1_S_1_), 97.00% (D_1_S_2_), 79.37% (D_1_S_3_), 115.51% (D_2_S_1_), 107.85% (D_2_S_2_), and 98.37% (D_2_S_3_).

On average, during the soybean growth period, the 10−30 cm soil layer in straw mulch treatments contained more water than the CK, with increases of 3.91% (D_1_S_1_), 4.75% (D_1_S_2_), 9.76% (D_1_S_3_), 3.45% (D_2_S_1_), 6.81% (D_2_S_2_), and 9.81% (D_2_S_3_). At the full-pod and full-seed stages, the soil water content at 10 cm under D_2_S_3_ was significantly higher than that under the CK (*p* < 0.05; Figure 4). In the 30−100 cm soil layer, straw mulching effects were less pronounced. Nevertheless, across the reproductive stages, the average soil water content in this layer increased by 1.21% (D_1_S_1_), 2.11% (D_1_S_2_), 4.77% (D_1_S_3_), 2.24% (D_2_S_1_), 2.39% (D_2_S_2_), and 5.18% (D_2_S_3_) compared with the CK.

### 3.2. Effect of Straw Mulching on Soil Temperature

The average temperature of the 0−25 cm soil layer increased steadily across all treatments as the reproductive period advanced (Table 1). From regreening to maturity, the soil temperature rose by 20.65 °C (CK), 21.23 °C (T_1_S_1_), 20.92 °C (T_1_S_2_), 20.22 °C (T_1_S_3_), 18.64 °C (T_2_S_1_), 18.96 °C (T_2_S_2_), and 16.35 °C (T_2_S_3_). The magnitude of increase ranged from 212% to 398.48%, with T_1_S_2_ showing the greatest warming effect and T_2_S_3_ the least (Table 1). The variation in the soil temperature (range and *C_v_*) exhibited a clear trend: both metrics increased, then decreased, and then increased again as the crop developed. The widest temperature range (4.24 °C) occurred at maturity, while the highest *C_v_* (17.31%) was observed at the regreening stage. Notably, the T_2_ treatments consistently showed lower *C_v_* values than the T_1_ treatments throughout the reproductive period, with T_2_S_3_ displaying the lowest variability—8.25% less than that of the CK (Table 1). Straw mulching significantly moderated the soil temperature. Across all reproductive stages, the T_1_S_1_, T_1_S_2_, T_1_S_3_, T_2_S_1_, T_2_S_2_, and T_2_S_3_ treatments reduced the average soil temperature in the 0–25 cm layer by 3.68%, 6.94%, 8.42%, 3.44%, 3.97%, and 10.85%, respectively, compared to the CK. The cooling effect was more pronounced with increasing straw cover thickness. Specifically, the T_1_S_2_ and T_1_S_3_ treatments significantly lowered the soil temperature from regreening to jointing (*p* < 0.05), but this effect diminished after the jointing stage. In contrast, the T_2_S_3_ treatment significantly reduced the soil temperature from jointing to maturity (*p* < 0.05; Table 1).

Straw mulching treatments had a distinct temperature-regulating effect that varied with the soybean growth stage (Table 2). During the higher temperature stages (fourth trifoliate leaf, full bloom, and full pod), straw mulching produced a clear cooling effect. The average soil temperature in the CK treatment during these stages was 32.28 °C, while the mulched treatments reduced soil temperatures by 0.34 °C to 4.73 °C. The cooling effect was strongest with increased straw thickness and gradually diminished as the reproductive period progressed. Specifically, from the fourth trifoliate leaf to full-pod stages, soil temperatures in the D_1_S_1_, D_1_S_2_, D_1_S_3_, D_2_S_1_, D_2_S_2_, and D_2_S_3_ treatments were reduced by 2.32%, 6.04%, 7.84%, 4.27%, 6.61%, and 8.07%, respectively, compared to in the CK. In contrast, during the later stages (full seed to full maturity), straw mulching exhibited a slight warming effect. The average soil temperature in the CK during this period was 20.88 °C, while the mulched treatments increased soil temperatures by 0.02 °C to 0.81 °C. This warming effect became more apparent as maturity approached but was less pronounced with thicker straw mulch.

During the entire soybean reproductive period, the average soil temperature in the 0−25 cm layer followed the order S_3_ < S_2_ < S_1_ < CK. Compared to the CK, the S_3_, S_2_, and S_1_ treatments lowered the average soil temperature by 5.12%, 4.02%, and 1.71%, respectively, with S_2_ and S_3_ showing statistically significant differences (*p* < 0.05; Table 2). At the fourth trifoliate leaf and full-bloom stages, the D_2_ treatments achieved a greater average cooling effect (2.04 °C) compared to the D_1_ treatments (1.74 °C), and temperature reductions under all straw mulching treatments were significantly different from that of the CK (*p* < 0.05). The range and *C_v_* of the soil temperature across treatments showed a trend of decreasing then increasing as the reproductive period progressed, with the largest range (4.73 °C) and *C_v_* (5.58%) observed at the fourth trifoliate leaf stage. This indicates greater sensitivity and variability in soil temperature regulation by straw mulching during early growth.

Throughout the reproductive period, the *C_v_* of soil temperature for treatments followed the order D_2_S_3_ < D_1_S_3_ < D_2_S_2_ < D_1_S_2_ < D_2_S_1_ < D_1_S_1_ < CK, indicating that thicker mulch provided the most stable soil temperatures (Table 2). Relative to the CK, the *C_v_* for the D_1_S_1_, D_1_S_2_, D_1_S_3_, D_2_S_1_, D_2_S_2_, and D_2_S_3_ treatments decreased by 10.78%, 19.79%, 19.79%, 22.39%, 15.99%, 19.62%, and 26.06%, respectively (Table 2). These findings demonstrate that straw mulching not only reduced the average soil temperature, particularly under higher thickness mulching, but also significantly minimized temperature fluctuations during the soybean reproductive period.

### 3.3. Effects of Straw Mulching on Soil Nutrients

**Figure 5:**
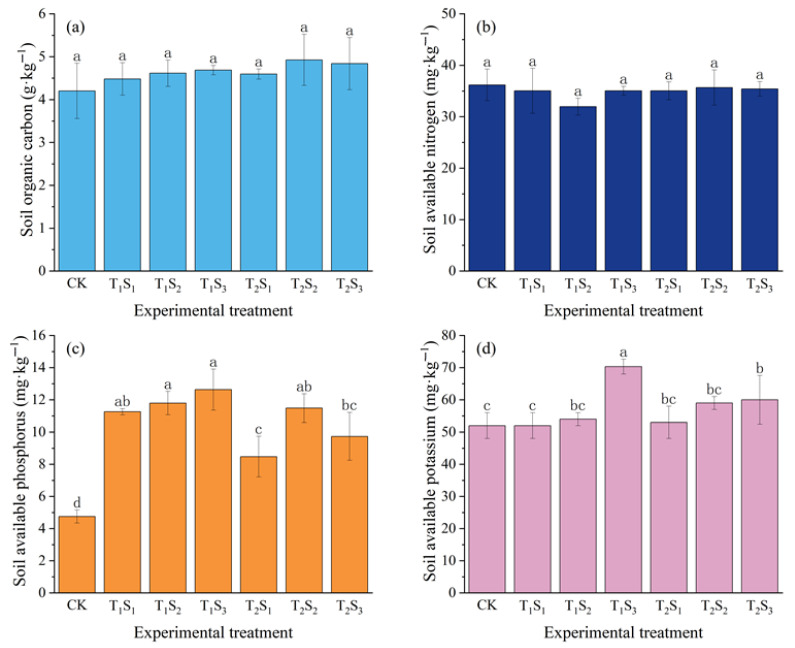
Effects of different straw mulching treatments on (**a**) soil organic carbon, (**b**) available nitrogen, (**c**) available phosphorus, and (**d**) available potassium in winter wheat soils. Different lowercase letters within the same column indicate significant differences between treatments (*p* < 0.05); treatments sharing the same lowercase letter are not significantly different (*p* > 0.05). CK: no mulching; T_1_S_1_/T_1_S_2_/T_1_S_3_: 1/3/5 cm crushed straw mulch applied since seedling stage; T_2_S_1_/T_2_S_2_/T_2_S_3_: 1/3/5 cm crushed straw mulch applied since jointing stage.

Straw mulching treatments significantly altered soil nutrient contents and storage in the 0–30 cm layer during the winter wheat growth period (Figure 5). Compared with the non-mulching control (CK), both seedling-stage (T_1_) and jointing-stage (T_2_) straw mulching increased soil organic carbon, available phosphorus, and available potassium. On average, T_1_ increased these nutrients by 9.37%, 150.28%, and 13.03%, while T_2_ increased them by 13.95%, 108.23%, and 10.26%, respectively. With increasing mulch thickness (S_1_, S_2_, S_3_), organic carbon, available phosphorus, and available potassium rose by 8.04−13.56%, 107.60−144.88%, and 0.96−25.32%, respectively, compared to the CK. However, straw mulching reduced soil available nitrogen by 1.41−11.69%. Notably, all straw mulching treatments significantly increased available phosphorus (*p* < 0.05), and the S_3_ treatments significantly increased available potassium (*p* < 0.05). The T_1_S_3_ treatment showed the greatest effect, raising available phosphorus and potassium by 165.89% and 34.6% over CK, respectively. In contrast, straw mulching had no significant effect on soil organic carbon or available nitrogen (*p* > 0.05).

**Figure 6:**
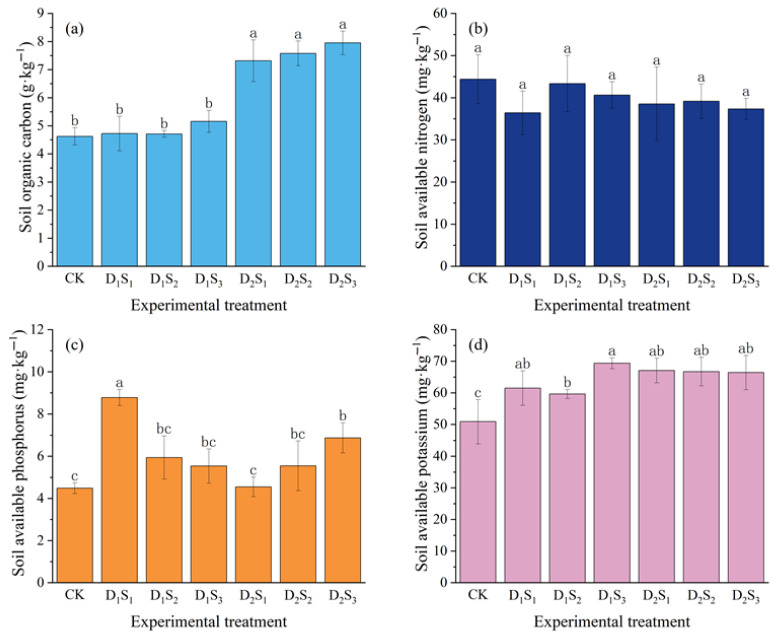
Effects of different straw mulching treatments on (**a**) soil organic carbon, (**b**) available nitrogen, (**c**) available phosphorus, and (**d**) available potassium in soybean soils. Different lowercase letters within the same column indicate significant differences between treatments (*p* < 0.05); treatments sharing the same lowercase letter are not significantly different (*p* > 0.05). CK: no straw mulching treatment; D_1_S_1_/D_1_S_2_/D_1_S_3_: seeding density of 1.8 × 10^5^ plants·ha^−1^ with straw mulch thickness of 1/3/5 cm; D_2_S_1_/D_2_S_2_/D_2_S_3_: seeding density of 3.6 × 10^5^ plants·ha^−1^ with straw mulch thickness of 1/3/5 cm.

During the soybean growth period, all the straw mulching treatments improved the soil organic carbon, available phosphorus, and available potassium contents in the 0−30 cm layer compared to in the no-mulched control (CK) (Figure 6). Specifically, low-density planting (D_1_) increased organic carbon, available phosphorus, and available potassium by 5.19%, 50.54%, and 24.78%, respectively, while high-density planting (D_2_) increased them by 64.67%, 26.05%, and 31.14%. With increasing mulch thickness (S_1_, S_2_, S_3_), soil organic carbon, available phosphorus, and available potassium rose by 30.17−41.74%, 28.07−48.53%, and 24.13−33.43%, respectively, compared to the CK. All straw mulching treatments, however, decreased available nitrogen by 2.36−18.00%. Straw mulching significantly increased the soil available potassium content across treatments (*p* < 0.05). Notably, high-density (D_2_) mulching significantly enhanced soil organic carbon (*p* < 0.05), with the greatest increase in D_2_S_3_ (organic carbon and available potassium increased by 72.08% and 30.52% over the CK, respectively). In D_1_ treatments, available phosphorus decreased with greater mulch thickness, while in D_2_ treatments, it increased, reaching 53.13% higher than the CK in D_2_S_3_. However, differences among the three high-density mulching treatments in available phosphorus and available nitrogen were not statistically significant (*p* > 0.05; Figure 6).

### 3.4. Effect of Straw Mulching on Crop Dry Matter, Yield, and Yield Components

#### 3.4.1. Effect of Straw Mulching on Crop Dry Matter

**Figure 7:**
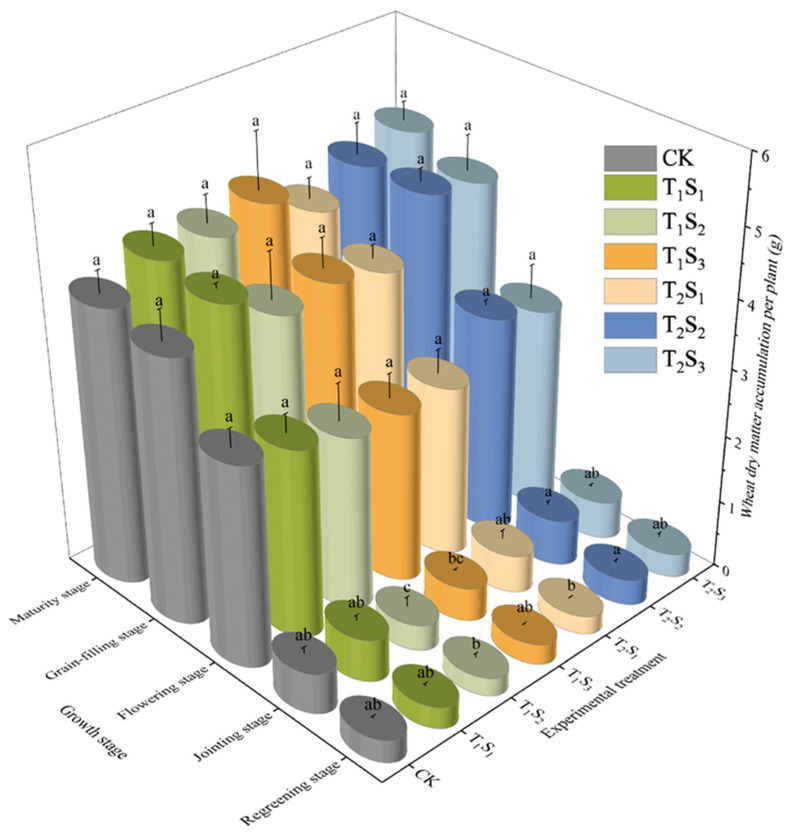
Changes in dry matter accumulation of winter wheat during the growth period under different straw mulching treatments. Different lowercase letters within the same column indicate significant differences between treatments (*p* < 0.05); treatments sharing the same lowercase letter are not significantly different (*p* > 0.05). CK: no mulching; T_1_S_1_/T_1_S_2_/T_1_S_3_: 1/3/5 cm crushed straw mulch applied since seedling stage; T_2_S_1_/T_2_S_2_/T_2_S_3_: 1/3/5 cm crushed straw mulch applied since jointing stage.

Dry matter accumulation per plant increased steadily throughout the growth period, accelerating markedly from the jointing to the grain-filling stage and peaking at maturity (Figure 7). Differences among treatments were minimal at the regreening and jointing stages but became increasingly apparent from flowering onwards. At maturity, dry matter accumulation per plant in the T_1_S_1_, T_1_S_2_, T_1_S_3_, T_2_S_1_, T_2_S_2_, and T_2_S_3_ treatments exceeded that in the CK by 8.17%, 8.29%, 12.25%, 1.90%, 10.48%, and 15.91%, respectively. The T_1_S_3_, T_2_S_2_, and T_2_S_3_ treatments achieved the greatest gains, particularly after the jointing stage. Within each growth stage, dry matter accumulation increased with greater straw mulching thickness. At maturity, the S_1_, S_2_, and S_3_ treatments increased the dry matter per plant by 5.04%, 9.39%, and 14.08% over the CK, respectively (Figure 7).

**Figure 8:**
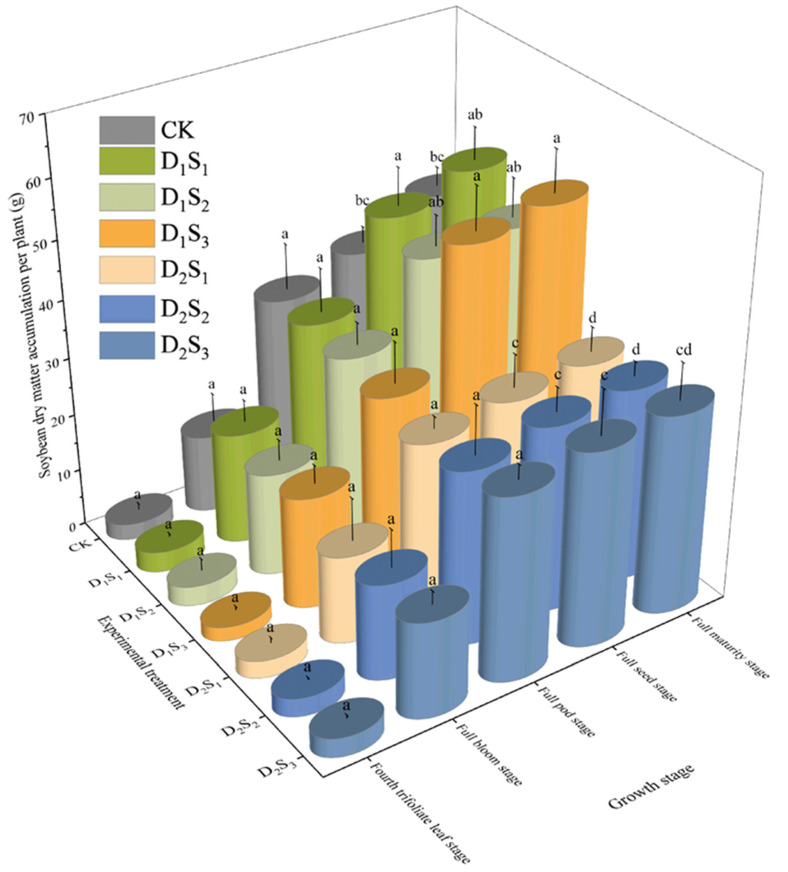
Changes in dry matter accumulation of soybeans during the growth period under different straw mulching treatments. Different lowercase letters within the same column indicate significant differences between treatments (*p* < 0.05); treatments sharing the same lowercase letter are not significantly different (*p* > 0.05). CK: no straw mulching treatment; D_1_S_1_/D_1_S_2_/D_1_S_3_: seeding density of 1.8 × 10^5^ plants·ha^−1^ with straw mulch thickness of 1/3/5 cm; D_2_S_1_/D_2_S_2_/D_2_S_3_: seeding density of 3.6 × 10^5^ plants·ha^−1^ with straw mulch thickness of 1/3/5 cm.

Dry matter accumulation per plant increased throughout the soybean growth period, with a rapid rise from the fourth trifoliate leaf to the full-seed stage and peaking at maturity (Figure 8). Early in the season (fourth trifoliate leaf to full-pod stage), straw mulching had a slight effect on dry matter accumulation compared to the CK, with increases ranging from 0.21 g to 0.78 g. However, from the full-seed stage to maturity, differences between treatments became pronounced, with dry matter accumulation per plant varying from −10.74 g to +11.19 g relative to that of the CK. This indicates a greater effect of straw mulching on dry matter accumulation during the later growth stages. At maturity, the D_1_S_1_, D_1_S_2_, and D_1_S_3_ treatments increased dry matter accumulation by 19.74%, 6.88%, and 25.91% compared to the CK, while D_2_S_1_, D_2_S_2_, and D_2_S_3_ treatments decreased it by 24.88%, 22.86%, and 20.46%, respectively. The D_1_ (low-density) treatments consistently achieved higher dry matter accumulation, especially after the full-seed stage, and this advantage became increasingly significant. Across all mulch thicknesses, D_1_ treatments outperformed D_2_, with dry matter at maturity in D_1_ treatments being 1.52 times higher than in D_2_ treatments. Notably, dry matter accumulation per plant for D_1_ treatments at the full-seed and maturity stages was significantly greater than that for D_2_ treatments (*p* < 0.05; Figure 8).

#### 3.4.2. Effects of Straw Mulching on Crop Yield and Their Components

The winter wheat yield was highest under the T_2_S_3_ treatment and lowest under T_1_S_3_, with a significant yield difference of 71.92% between these treatments (*p* < 0.05; Table 3). On average, T_2_ treatments produced a yield of 7132.48 kg·ha^−1^, representing a 23.83% increase over that of the CK, while T_1_ treatments averaged 5366.19 kg·ha^−1^, a 1.22% decrease compared to that of the CK. Straw mulching at the seedling stage (T_1_) did not significantly affect the yield relative to the CK (*p* > 0.05), and increasing mulching thickness tended to reduce the yield. Compared with the CK, T_1_S_1_ increased the yield by 16.43%, while T_1_S_2_ and T_1_S_3_ reduced the yield by 4.71% and 15.40%, respectively, indicating that a thinner mulch layer was more beneficial at this stage. In contrast, all T_2_ (jointing stage) mulching treatments increased the wheat yield, with T_2_S_1_, T_2_S_2_, and T_2_S_3_ yields surpassing the CK by 16.84%, 31.58%, and 45.45%, respectively. The yield increases in T_2_S_2_ and T_2_S_3_ were statistically significant (*p* < 0.05). Overall, straw mulching at the jointing stage, particularly with greater mulch thickness, significantly promoted the winter wheat yield, while excessive mulch at the seedling stage hindered yield formation.

Straw mulching had no significant effect on the wheat thousand-grain weight, though all mulched treatments showed increases of 3.48–10.80% compared to the CK. For the spike density, T_1_ treatments were not significantly different from the CK (*p* > 0.05): T_1_S_1_ and T_1_S_2_ increased the spike density by 9.50% and 9.24%, respectively, while the T_1_S_3_ treatment reduced it by 5.14% (Table 3). In contrast, the T_2_ mulching treatments, particularly T_2_S_2_ and T_2_S_3_, significantly increased the spike density by 28.62% and 34.02%, respectively (*p* < 0.05). T_2_S_1_ increased the spike density by 11.81% over that of the CK, but this was not statistically significant. Regarding the grain number per spike, only T_2_S_3_ resulted in a significant increase of 15.01% compared to the CK (*p* < 0.05), while other treatments showed non-significant increases ranging from 0.68% to 5.9% (Table 3).

The soybean yield was highest in the D_2_S_2_ treatment and lowest in the CK, with a significant yield difference of 50.31% (*p* < 0.05; Table 4). The average yield for the D_1_ treatments was 3059.51 kg·ha^−1^, while the D_2_ treatments averaged 3361.23 kg·ha^−1^—representing increases of 23.99% and 36.22% over the CK, respectively. For the D_1_ treatments, the yield increased progressively with a greater straw mulching thickness: D_1_S_1_, D_1_S_2_, and D_1_S_3_ increased yields by 17.86%, 25.87%, and 28.25% compared to the CK, though these differences were not statistically significant (*p* > 0.05). In contrast, all D_2_ treatments produced higher yields, with D_2_S_1_, D_2_S_2_, and D_2_S_3_ increasing yields by 28.55%, 50.31%, and 29.80% over the CK, respectively; the increase in D_2_S_2_ was statistically significant (*p* < 0.05).

In the D_1_ treatments, the number of pods per plant increased progressively with a greater straw mulching thickness, reaching its highest value in the D_1_S_3_ treatment (Table 4). In contrast, the D_2_ treatments showed a decrease in pods per plant by 8.08% to 20.23% compared with the CK. All straw mulching treatments resulted in higher hundred-grain weights than the CK. Specifically, the hundred-grain weights in the D_1_S_1_, D_1_S_2_, D_1_S_3_, D_2_S_1_, D_2_S_2_, and D_2_S_3_ treatments increased by 3.47%, 4.62%, 5.14%, 3.31%, 7.89%, and 2.49%, respectively, over the CK. The highest value was observed in D_2_S_2_. However, differences in the hundred-grain weight among treatments were not statistically significant (*p* > 0.05; Table 4).

### 3.5. Crop Yield Drivers

#### 3.5.1. Wheat Yield Drivers

**Figure 9:**
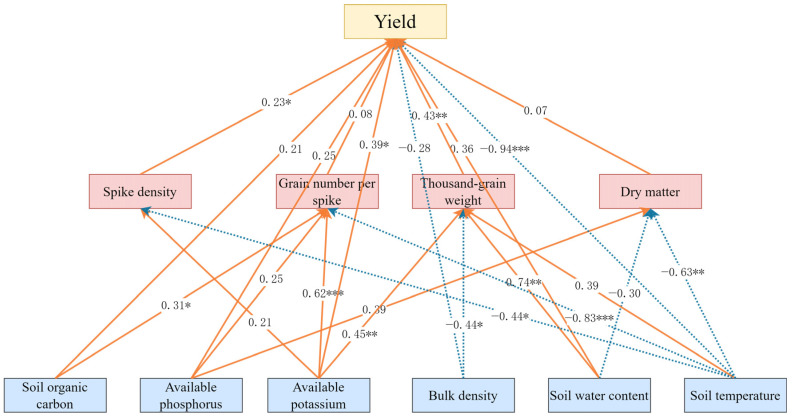
Wheat structural equation model path diagram. Solid lines indicate positive effects, dashed lines represent negative effects, and the path coefficients are provided along the paths. Asterisks denote statistical significance: * *p* < 0.05, ** *p* < 0.01, *** *p* < 0.001.

To identify the key drivers of the wheat yield under straw mulching, a structural equation model (SEM) was constructed to link the soil physicochemical properties, yield components, and yield. Soil available nitrogen was excluded from the final model due to its minimal contribution. The optimized SEM demonstrated excellent fit (χ^2^/df = 1.076 < 3.00, CFI = 0.982 > 0.90, RMSEA = 0.062 < 0.08), with an explanatory power (R^2^) of 0.850 for the wheat yield (Figure 9). The model revealed that both the amount and timing of straw mulching significantly influenced soil organic carbon, available phosphorus, available potassium, bulk density, water content, and soil temperature. These soil property changes affected the wheat yield either directly or indirectly via yield components. Specifically, increases in soil organic carbon, available potassium, available phosphorus, and water content directly enhanced the wheat yield, while higher soil bulk density and temperature directly inhibited the yield. Additionally, soil temperature and available potassium indirectly affected the spike density, grain number per plant, thousand-grain weight, and dry matter content, further promoting the yield. Among all factors, soil temperature exhibited the strongest total effect on the wheat yield, followed by available potassium, water content, bulk density, available phosphorus, and organic carbon (Table 5).

#### 3.5.2. Soybean Yield Drivers

**Figure 10:**
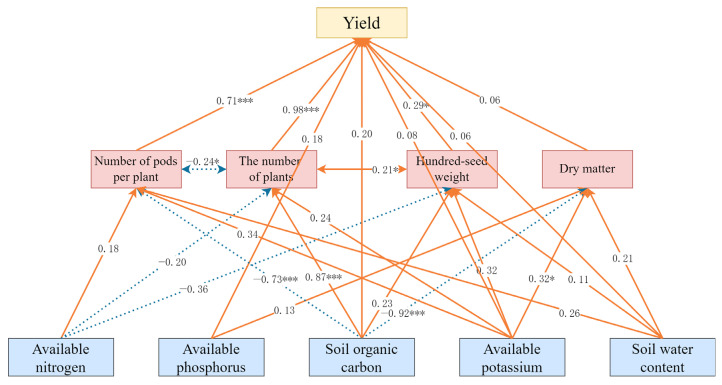
Path diagram of the soybean structural equation model. Solid lines represent positive effects, dashed lines indicate negative effects, and the path coefficients are provided along the paths. Asterisks denote statistical significance: * *p* < 0.05, *** *p* < 0.001.

Soil temperature and bulk density were excluded from the final soybean structural equation model due to their negligible contribution to yield. The optimized SEM demonstrated good fit (χ^2^/df = 1.100 < 3.00, CFI = 0.979 > 0.90, RMSEA = 0.071 < 0.08), with an explanatory power (R^2^) of 0.788 for the soybean yield (Figure 10). Under varying planting densities and straw mulching rates, changes in the soil physicochemical properties and yield components directly or indirectly affected the soybean yield. The straw mulch amount and planting density significantly influenced soil organic carbon, available nitrogen, available phosphorus, available potassium, and water content, which in turn impacted the yield either directly or via yield components. Specifically, increased soil water content, available potassium, available phosphorus, and organic carbon directly promoted the soybean yield, whereas higher available nitrogen had an inhibitory effect. Available potassium and organic carbon also indirectly enhanced the yield by increasing the effective number of plants, pods per plant, hundred-grain weight, and dry matter accumulation. Overall, the total effects of the measured factors on the soybean yield ranked as follows: available potassium > organic carbon > water content > available phosphorus > available nitrogen (Table 6).

## 4. Discussion

### 4.1. Effects of Straw Return Patterns on Soil Water, Nutrients, and Temperature

Straw return practices substantially influence soil environmental quality by modulating soil water content, nutrient availability, and temperature regimes. In this study, we observed that straw mulching consistently improved the soil’s physical and chemical properties, thereby creating a more favorable environment for crop growth. Our findings demonstrate that straw mulching enhances soil water retention, especially in the primary root zone (0–30 cm), with increases ranging from 3.45% to 9.81% compared to that of the control. This improvement is primarily attributable to the straw layer acting as a physical barrier, which suppresses soil evaporation and limits direct soil–atmosphere interactions. Thicker straw mulch amplified this effect, supporting the notion that increased mulch thickness more effectively conserves soil moisture. These results align with prior studies reporting similar benefits of straw mulching for water retention across various crop systems [32,33,34].

Straw mulching also played a pivotal role in regulating soil temperature throughout the crop growth period. Across all treatments and growth stages, we observed a consistent cooling effect, with temperature reductions proportional to the mulch thickness. The straw’s insulating properties were especially beneficial during periods of high ambient temperature, as the mulch layer reduced heat absorption and promoted evaporative cooling, mitigating heat stress on crops. Conversely, during cooler periods, straw mulch reduced heat loss and provided thermal insulation, resulting in slightly higher nighttime soil temperatures. This dual role highlights straw mulching’s capacity to buffer soil temperature fluctuations and promote more stable microclimatic conditions [35,36,37,38,39,40]. Notably, the increased coefficient of variation (*C_v_*) in the soil temperature under mulching suggests enhanced diurnal and vertical thermal gradients, which may further influence soil biological activity and nutrient cycling.

In addition to physical improvements, straw return significantly enhanced the soil nutrient status, with increases in organic carbon, available phosphorus, and available potassium. The incorporation of organic matter through straw addition directly elevated soil organic carbon, while the decomposition process, facilitated by microbial activity, mobilized phosphorus and potassium, increasing their availability for plant uptake [41,42]. However, a notable decline in soil available nitrogen was observed, particularly under wheat–soybean rotation. This reduction can be explained by microbial immobilization, as microbes utilize soil nitrogen for decomposing high C/N ratio straw, temporarily reducing nitrogen availability in the soil [43,44,45]. Furthermore, the dynamics of available phosphorus varied with plant density and mulch thickness. Lower phosphorus levels under sparse plant density may result from reduced root exudation and increased microbial competition for nutrients, whereas denser root systems under higher plant density promoted phosphorus solubilization and availability [46,47].

Taken together, our results indicate that straw mulching exerts multifaceted effects on soil environment quality. By simultaneously improving soil water retention, regulating temperature, and enhancing nutrient availability, straw return supports robust crop growth and resilience to abiotic stress. However, these benefits must be balanced against potential drawbacks, such as reduced nitrogen availability due to microbial immobilization, particularly in the early stages of straw decomposition. Understanding these interactive effects is crucial for optimizing straw return practices to achieve sustainable productivity and soil health.

### 4.2. Effects of Straw Return Patterns on Crop Dry Matter Accumulation, Yield, and Yield Components

This study highlights the complex role of straw return patterns in influencing dry matter accumulation, crop yields, and their components in both winter wheat and soybean systems. Our findings indicate that straw mulching generally benefits final dry matter accumulation and yield, but the magnitude and direction of these effects depend on factors such as mulch thickness, application timing, and planting density. Across all treatments, straw mulching consistently enhanced the final dry matter accumulation at crop maturity compared to the control (CK). This trend, which aligns with the observations of Yang et al. [48], can be attributed to the improved soil moisture retention and stabilized thermal conditions provided by straw cover during critical growth periods. Enhanced water availability and moderate soil temperatures promote sustained photosynthetic activity and assimilate translocation, supporting continuous dry matter synthesis. Furthermore, the gradual decomposition of straw enriches the soil with essential nutrients such as carbon, phosphorus, and potassium, thereby boosting soil fertility and further supporting crop growth [49]. However, the effect of straw return on dry matter accumulation varied between crops and planting densities. In soybeans, high-density (D_2_) treatments resulted in significantly lower dry matter accumulation than low-density (D_1_) treatments. The greater accumulation in D_1_ can be attributed to reduced inter-plant competition, allowing individual plants better access to water, nutrients, and light. This supports enhanced leaf expansion and photosynthetic efficiency, ultimately favoring dry matter production. In contrast, high-density planting intensified competition, constrained individual plant growth, and limited photosynthetic capacity [50].

The impact of straw mulching on crop yields was closely tied to the timing and thickness of application, as well as planting density. A moderate straw mulch application (1 cm) at the seedling stage led to a 16.43% increase in the wheat yield compared to the control, demonstrating the yield-promoting effect of an appropriate mulch layer. However, excessive mulching (3 cm or 5 cm) at this stage resulted in yield reductions of 4.71% and 15.40%, respectively. This suggests that while thin straw mulch can enhance early wheat growth by improving soil moisture and nutrient availability, excessive mulching may reduce soil aeration and lower soil temperature, impeding root development and nutrient uptake. Moreover, overly thick mulch may create shading and excessive humidity, potentially increasing disease risk and inhibiting seedling establishment, which ultimately suppresses yield formation [51,52,53]. Interestingly, when straw mulching was applied later, at the jointing stage, even higher mulch levels resulted in improved yields and yield components, including increases in the spike density, grains per spike, and thousand-grain weight (by 11.81–34.02%, 0.77–15.01%, and 3.47–10.78%, respectively, compared with the CK). This can be explained by the fact that mulching at the jointing stage avoids negative effects on early seedling establishment and instead supports plant growth during a period of high demand for water and nutrients. The mulch conserves soil moisture and stabilizes temperature, providing a favorable microenvironment for root development and nutrient uptake, thereby promoting both vegetative and reproductive growth [54].

For soybeans, straw mulching treatments generally led to higher yields than the control, mainly by enhancing soil moisture retention, moderating temperature fluctuations, and supplying nutrients through straw decomposition [55]. However, the response varied with planting density. In high-density (D_2_) treatments, while plant numbers per area increased, the number of pods per plant declined (by 8.08–20.23%), reflecting intensified competition for limited resources and a consequent reduction in assimilate partitioning and seed development [56]. In low-density (D_1_) treatments, the soybean yield increased with the mulch thickness, reaching a maximum under D_1_S_3_ (3164.60 kg·ha^−1^). In contrast, under high-density planting, the maximum yield (3708.93 kg·ha^−1^) was observed at 3 cm mulch. These patterns suggest that, at a low density, thicker mulch can effectively conserve soil moisture and regulate temperature, thus supporting better root and shoot development [57]. However, in high-density stands, excessive mulch may impede soil aeration, restrict root respiration, and limit nutrient uptake, offsetting the benefits of moisture conservation and ultimately reducing yield [58].

Overall, the effects of straw return on crop dry matter accumulation and yield are strongly context-dependent. Optimal benefits are achieved by carefully balancing mulch thickness, application timing, and planting density to match the physiological needs of the crop at different developmental stages. While straw mulching can significantly improve soil quality and promote crop productivity, excessive mulch or inappropriate application timing may undermine these advantages by disrupting the soil–plant microenvironment. These findings underscore the need for site-specific straw management strategies to maximize the agronomic and ecological benefits of straw return in crop production systems.

### 4.3. Drivers of Wheat and Soybean Yield Under Straw Return

This study utilized structural equation modeling to elucidate the primary factors influencing wheat and soybean yields under straw return management. The resulting models accounted for a substantial proportion of yield variability—explaining 85.00% of the wheat yield and 78.80% of the soybean yield variation—demonstrating that crop productivity is shaped by multiple, interacting drivers. For wheat, the model identified soil water content and available potassium as the most significant positive contributors to yield (Table 5). These results are consistent with prior research [59], which established that straw mulching elevates soil moisture and boosts dryland wheat yields. Enhanced water availability and potassium directly support vital physiological processes, such as sustained photosynthesis and grain-filling, leading to higher grain number per spike and increased thousand-grain weight. Additionally, soil organic carbon was found to positively influence yield and its components, further underscoring the value of improved soil fertility under straw return systems. Higher organic carbon levels support nutrient cycling and root growth, both critical for yield formation. However, the model also revealed that increases in soil bulk density have a significant negative effect on the thousand-grain weight. Elevated bulk density, indicative of soil compaction, restricts root penetration and limits both water and nutrient uptake, ultimately reducing the flow of photosynthates to developing seeds during crucial reproductive stages. Moreover, higher soil temperatures were associated with reduced yield and its components. Excessive soil temperatures can impair root activity, shorten the reproductive period, and curtail the accumulation of photosynthetic products, thereby negatively impacting the final yield [60].

In the case of soybeans, soil organic carbon emerged as the most important positive driver of yield (Table 6), corroborating findings by Akhtar et al. [61]. Improved organic carbon content enhances soil structure, promotes root development, and facilitates nutrient uptake—all of which contribute to increased yield. Notably, the present study observed that while elevated soil organic carbon can sometimes suppress dry matter accumulation and the number of pods per plant, it simultaneously increases the effective plant number and hundred-grain weight. This suggests that the moderate enrichment of soil organic carbon improves certain yield components, even if excessive levels may occasionally disrupt nutrient balance or reduce soil permeability. The analysis further revealed that the effective number of plants was more strongly correlated with the overall soybean yield than either the number of pods per plant or dry matter per plant. This highlights the pivotal role of stand establishment and plant survival under straw mulching in determining the final yield outcomes. Lastly, the study identified available (fast-acting) potassium as a crucial factor promoting dry matter accumulation in soybeans. Potassium ions play essential roles in activating enzymes involved in photosynthesis and carbohydrate metabolism, thereby supporting increased photosynthetic rates, starch synthesis, and, ultimately, dry matter production [62].

In summary, the drivers of wheat and soybean yield under straw return systems are multifaceted and crop-specific. For wheat, optimizing soil water and potassium availability, while avoiding compaction and excessive soil temperatures, is critical for maximizing yield. For soybeans, enhancing soil organic carbon within optimal limits and ensuring adequate potassium supply are key strategies. Collectively, these results highlight the importance of tailored soil and crop management practices under straw return regimes to fully realize yield benefits in different cropping systems.

## 5. Conclusions

This study systematically evaluated the effects of varying straw mulching rates on soil water content, temperature regulation, nutrient dynamics, and crop yields in a winter wheat–soybean rotation system. The core findings are as follows: (1) Straw mulching significantly improved soil moisture, particularly in the 0–30 cm layer, with the effect intensifying alongside increased mulch thickness. Mulching moderated soil temperature in a bidirectional manner: it reduced temperature during early and mid-growth stages and promoted warming in late stages, with the magnitude of cooling positively related to the mulch amount. (2) Mulching substantially enhanced soil organic carbon, available phosphorus, and potassium, while slightly decreasing available nitrogen, suggesting a shift in nutrient transformation pathways and emphasizing the need for balanced nitrogen management. (3) The yield benefits from straw mulching were crop- and stage-specific. In winter wheat, moderate mulching at the jointing stage (T_2_) led to the highest yield improvement (up to 45.45%), while excessive early mulching reduced yield, indicating a threshold effect. For soybeans, all mulching treatments increased the yield, with the optimal effect observed under medium mulching combined with high-density planting, resulting in a 50.31% yield increase. (4) Path analysis revealed that increases in soil water content, available potassium, phosphorus, and organic carbon were key drivers of yield improvement in both crops, while excessive soil temperature and bulk density limited wheat yields. Notably, elevated soil available nitrogen showed a negative correlation with soybean yields, highlighting crop-specific nutrient requirements. These findings demonstrate that rational straw mulching, tailored to crop type, development stage, and planting density, can significantly enhance resource use efficiency, optimize soil microenvironment, and increase the productivity of rainfed rotation systems. The study provides a theoretical basis for the sustainable intensification of cereal–legume rotations in rainfed regions.

Despite these advances, several areas warrant further investigation: (1) The observed reduction in available nitrogen under mulching underscores the need for in-depth studies on nitrogen transformation and its long-term impact on soil fertility. (2) Future research should involve multi-year, multi-site trials to validate the sustainability and generalizability of the recommended mulching strategies. (3) Exploration of the synergistic effects of straw mulching with other soil management and fertilization techniques is necessary to maximize system resilience and yield stability.

## Figures and Tables

**Table 1:** Mean temperature (°C) of the 0−25 cm soil layer during different growth stages of winter wheat.

Experimental Treatment	Growth Stage					Average	*C_v_* (%)
	Regreening Stage	Jointing Stage	Flowering Stage	Grain-Filling Stage	Maturity Stage		
CK	7.64 ± 0.52 a	16.40 ± 0.55 a	20.40 ± 0.91 a	22.97 ± 1.55 a	28.29 ± 1.31 a	19.14 a	40.48%
T_1_S_1_	6.21 ± 0.31 b	15.96 ± 0.06 ab	20.01 ± 0.61 a	22.22 ± 0.43 a	27.44 ± 0.69 ab	18.37 ab	43.34%
T_1_S_2_	5.25 ± 0.40 c	15.36 ± 0.75 b	20.10 ± 0.66 a	21.79 ± 0.72 a	26.16 ± 0.86 bc	17.73 bcd	45.01%
T_1_S_3_	5.39 ± 0.49 bc	14.39 ± 0.40 c	19.88 ± 0.74 a	21.54 ± 0.96 a	25.60 ± 0.80 c	17.36 cd	44.97%
T_2_S_1_	7.84 ± 0.55 a	15.87 ± 0.24 ab	19.82 ± 0.44 a	21.89 ± 0.52 a	26.48 ± 0.31 bc	18.38 ab	38.24%
T_2_S_2_	7.65 ± 0.60 a	15.21 ± 0.43 b	19.95 ± 0.37 a	21.93 ± 0.49 a	26.61 ± 0.41 bc	18.27 abc	39.49%
T_2_S_3_	7.71 ± 0.57 a	13.98 ± 0.15 c	18.55 ± 0.40 b	20.09 ± 0.51 b	24.05 ± 0.29 d	16.87 d	37.14%
Range	2.59	2.42	1.85	2.88	4.24		
*C_v_* (%)	17.31%	5.89%	3.86%	4.96%	5.40%		

Note: Different lowercase letters within the same column indicate significant differences between treatments (*p* < 0.05); treatments sharing the same lowercase letter are not significantly different (*p* > 0.05). *C_v_* is the coefficient of variation, which represents the degree of dispersion of the average temperature in the 0−25 cm soil layer either within the same treatment across different growth stages, or across different treatments at the same growth stage. CK: no mulching; T_1_S_1_/T_1_S_2_/T_1_S_3_: 1/3/5 cm crushed straw mulch applied since seedling stage; T_2_S_1_/T_2_S_2_/T_2_S_3_: 1/3/5 cm crushed straw mulch applied since jointing stage.

**Table 2:** Mean soil temperatures (°C) in the 0−25 cm layer at different growth stages of soybean.

Experimental Treatment	Growth Stage					Average	*C_v_* (%)
	Fourth Trifoliate Leaf Stage	Full-Bloom Stage	Full-Pod Stage	Full-Seed Stage	Full-Maturity Stage		
CK	35.63 ± 1.40 a	32.00 ± 0.70 a	29.20 ± 0.54 ab	22.09 ± 1.27 a	19.67 ± 0.73 a	27.71 a	24.21%
D_1_S_1_	33.90 ± 0.35 ab	31.30 ± 0.66 ab	29.38 ± 0.39 a	22.38 ± 0.35 a	20.09 ± 0.49 a	27.41 ab	21.60%
D_1_S_2_	31.90 ± 0.69 cd	30.40 ± 0.44 cd	28.68 ± 0.36 ab	22.34 ± 0.45 a	20.09 ± 0.26 a	26.68 bc	19.42%
D_1_S_3_	31.13 ± 0.32 d	29.63 ± 0.32 d	28.48 ± 0.05 b	22.13 ± 0.34 a	19.92 ± 0.39 a	26.26 c	18.79%
D_2_S_1_	33.27 ± 0.65 bc	30.57 ± 0.21 bc	28.86 ± 0.16 ab	22.26 ± 0.51 a	20.41 ± 0.31 a	27.06 abc	20.34%
D_2_S_2_	31.80 ± 1.28 cd	30.03 ± 0.31 cd	28.60 ± 0.20 b	21.95 ± 0.67 a	20.14 ± 0.47 a	26.51 bc	19.46%
D_2_S_3_	30.90 ± 1.48 d	29.63 ± 0.45 d	28.49 ± 0.61 b	22.11 ± 0.53 a	20.48 ± 0.58 a	26.32 c	17.90%
Range	4.73	2.37	0.90	0.43	0.81		
*C_v_* (%)	5.58%	3.00%	1.60%	2.48%	2.41%		

Note: Different lowercase letters within the same column indicate significant differences between treatments (*p* < 0.05); treatments sharing the same lowercase letter are not significantly different (*p* > 0.05). *C_v_* is the coefficient of variation, which represents the degree of dispersion of the average temperature in the 0−25 cm soil layer either within the same treatment across different growth stages, or across different treatments at the same growth stage. CK: no straw mulching treatment; D_1_S_1_/D_1_S_2_/D_1_S_3_: seeding density of 1.8 × 10^5^ plants·ha^−1^ with straw mulch thickness of 1/3/5 cm; D_2_S_1_/D_2_S_2_/D_2_S_3_: seeding density of 3.6 × 10^5^ plants·ha^−1^ with straw mulch thickness of 1/3/5 cm.

**Table 3:** Winter wheat yield and its components under different straw mulching treatments.

Experimental Treatment	Yield (kg·ha^−1^)	Spike Density (Spikes·m^−2^)	Grain Number per Spike	Thousand-Grain Weight (g)
CK	5432.70 ± 1088.25 cd	259.67 ± 27.65 b	41.03 ± 2.22 bc	46.58 ± 0.44 a
T_1_S_1_	6325.47 ± 447.40 bc	284.33 ± 24.34 ab	43.45 ± 1.30 b	51.11 ± 2.13 a
T_1_S_2_	5176.93 ± 98.73 cd	283.67 ± 61.16 ab	41.31 ± 0.31 bc	50.65 ± 1.57 a
T_1_S_3_	4596.17 ± 736.67 d	246.33 ± 27.79 b	38.42 ± 1.43 c	50.05 ± 3.78 a
T_2_S_1_	6347.30 ± 712.58 bc	290.33 ± 11.85 ab	41.35 ± 0.69 bc	48.20 ± 0.91 a
T_2_S_2_	7148.20 ± 754.63 ab	334.00 ± 30.20 a	41.48 ± 3.36 bc	50.16 ± 2.51 a
T_2_S_3_	7901.93 ± 780.57 a	348.00 ± 45.21 a	47.19 ± 0.28 a	51.61 ± 5.49 a

Note: Different lowercase letters within the same column indicate significant differences between treatments (*p* < 0.05); treatments sharing the same lowercase letter are not significantly different (*p* > 0.05). CK: no mulching; T_1_S_1_/T_1_S_2_/T_1_S_3_: 1/3/5 cm crushed straw mulch applied since seedling stage; T_2_S_1_/T_2_S_2_/T_2_S_3_: 1/3/5 cm crushed straw mulch applied since jointing stage.

**Table 4:** Soybean yield and its components under different straw mulching patterns.

Experimental Treatment	Yield (kg·ha^−1^)	Number of Pods per Plant	Hundred-Grain Weight (g)
CK	2467.50 ± 526.08 b	30.94 ± 4.83 abc	30.53 ± 1.71 a
D_1_S_1_	2908.20 ± 232.02 ab	31.32 ± 3.29 abc	31.59 ± 3.04 a
D_1_S_2_	3105.73 ± 407.88 ab	35.47 ± 2.02 ab	31.94 ± 0.73 a
D_1_S_3_	3164.60 ± 369.04 ab	38.05 ± 10.39 a	32.10 ± 1.69 a
D_2_S_1_	3171.97 ± 16.61 ab	24.68 ± 1.57 c	31.54 ± 2.02 a
D_2_S_2_	3708.93 ± 741.47 a	28.44 ± 3.63 abc	32.94 ± 2.79 a
D_2_S_3_	3202.80 ± 341.50 ab	25.51 ± 5.26 bc	31.29 ± 0.57 a

Note: Different lowercase letters within the same column indicate significant differences between treatments (*p* < 0.05); treatments sharing the same lowercase letter are not significantly different (*p* > 0.05). CK: no straw mulching treatment; D_1_S_1_/D_1_S_2_/D_1_S_3_: seeding density of 1.8 × 10^5^ plants·ha^−1^ with straw mulch thickness of 1/3/5 cm; D_2_S_1_/D_2_S_2_/D_2_S_3_: seeding density of 3.6 × 10^5^ plants·ha^−1^ with straw mulch thickness of 1/3/5 cm.

**Table 5:** Relative size of drivers of wheat yield.

Parameters	Soil Temperature	Soil Water Content	Bulk Density	Available Potassium	Available Phosphorus	Soil Organic Carbon
Total effect	−0.853	0.657	−0.465	0.675	0.300	0.236
Direct effect	−0.944	0.361	−0.278	0.385	0.254	0.212
Indirect effect	0.091	0.296	−0.187	0.290	0.046	0.024

**Table 6:** Relative size of drivers of soybean yield.

Parameters	Soil Water Content	Available Potassium	Available Phosphorus	Available Nitrogen	Soil Organic Carbon
Total effect	0.288	0.670	0.190	−0.164	0.548
Direct effect	0.061	0.082	0.183	0.000	0.201
Indirect effect	0.227	0.588	0.007	−0.164	0.347

## Data Availability

The data that support this study cannot be publicly shared due to ethical or privacy reasons and may be shared upon reasonable request to the corresponding author if appropriate.
